# Psychedelics, Mystical Experience, and Therapeutic Efficacy: A Systematic Review

**DOI:** 10.3389/fpsyt.2022.917199

**Published:** 2022-07-12

**Authors:** Kwonmok Ko, Gemma Knight, James J. Rucker, Anthony J. Cleare

**Affiliations:** ^1^Centre for Affective Disorders, Institute of Psychiatry, Psychology and Neuroscience, King's College London, London, United Kingdom; ^2^National Institute for Health Research Biomedical Research Centre, South London and Maudsley NHS Foundation Trust, King's College London, London, United Kingdom; ^3^South London and Maudsley NHS Foundation Trust, Bethlem Royal Hospital, Beckenham, United Kingdom

**Keywords:** psychedelic therapy, mystical experience, psilocybin, ayahuasca, ketamine, cancer-related distress, substance use disorder (SUD), depressive disorders

## Abstract

The mystical experience is a potential psychological mechanism to influence outcome in psychedelic therapy. It includes features such as oceanic boundlessness, ego dissolution, and universal interconnectedness, which have been closely linked to both symptom reduction and improved quality of life. In this review, 12 studies of psychedelic therapy utilizing psilocybin, ayahuasca, or ketamine were analyzed for association between mystical experience and symptom reduction, in areas as diverse as cancer-related distress, substance use disorder, and depressive disorders to include treatment-resistant. Ten of the twelve established a significant association of correlation, mediation, and/or prediction. A majority of the studies are limited, however, by their small sample size and lack of diversity (gender, ethnic, racial, educational, and socioeconomic), common in this newly re-emerging field. Further, 6 out of 12 studies were open-label in design and therefore susceptible to bias. Future studies of this nature should consider a larger sample size with greater diversity and thus representation by use of randomized design. More in-depth exploration into the nature of mystical experience is needed, including predictors of intensity, in order to maximize its positive effects on treatment outcome benefits and minimize concomitant anxiety.

**Systematic Review Registration:** PROSPERO, identifier CRD42021261752.

## Introduction

The application of psychedelic therapy for a range of psychiatric illnesses is undergoing examination (1, 2). Though studied in the 1960s and mid-70s, there was then a gap in study until the mid-1990s due to controversy and legal prohibition. As the evidence to date has demonstrated broad clinical potential for antidepressant and anxiolytic effects (3), PTSD (4), addiction (5), end-of-life distress (6), and potentially others, psychedelic therapy is emerging as an alternative to the current standard treatments. To date, studies indicate not only effectiveness but also safety, with fewer potential side effects than other forms of medication (7, 8). Since medication side effects and non-adherence are problems in mental health treatment (9), the intermittent nature of psychedelic therapy may be more acceptable to patients, resulting in increased compliance. It may also provide symptomatic relief to those who have had minimal response or have developed a resistance to currently accepted psychiatric medications.

Both neurobiological and psychological mechanisms related to psychedelics have been identified. These include emotional breakthrough (10), increased psychological flexibility (11), and mystical experience (12) to include oceanic boundlessness [OBN], universal interconnectedness, ego dissolution, and transcendence of time and space (13). OBN can be defined as the boundlessness of self and/or feeling of being one with the universe; the 4 sub-factors of OBN in ASC questionnaires are insightfulness, blissful state, experience of unity, and spiritual experience (14). Ego dissolution refers to reduction or loss of one's sense of personal identity; this can be positive, in the form of OBN (experience of unity), or negative, in producing anxiety (15).

Personally meaningful insight may be elicited by both psychedelics and mystical experience, and as a primary goal of psychotherapy, thus may be closely related to therapeutic outcome (16). Hayes et al. (17) defines psychological flexibility as ‘persisting or changing behavior in the service of chosen values' (p. 7). It has been further explored by Watts and Luoma (18) in the context of Acceptance and Commitment Therapy and the ACE model (Accept, Connect, and Embody), which postulates that the fundamental psychotherapeutic mechanism of psychedelics is in the facilitation of reinforcement of psychological flexibility. Hendricks (19) expanded the mystical experience to include the emotion of “awe” that arose from such experience, which he equated to “openness” in personality trait theory. Based on classic research in the 1950s to 1970s that indicated a low response to treatment in patients with rigid personality traits, he further proposed that this is the fundamental characteristic of mystical experience and a potential catalyst for long-term therapeutic outcome.

Psychedelics commonly used for therapeutic purposes include lysergic acid diethylamide [LSD], psilocybin, mescaline, and ayahuasca. 3,4-methylenedioxymethamphetamine [MDMA] and ketamine are also being studied for similar application, though they do not strictly fall into the category of psychedelic drugs; the former is classified as an amphetamine, while the latter is most frequently described as a dissociative anesthetic. Ketamine in particular has strong psychedelic features (20). All have induction of mystical experience as a shared feature, including MDMA (21, 22) and ketamine (23, 24).

Mystical experience may have a relationship to psychedelic therapy outcome as some studies have indicated an application of the former may result in ‘abrupt, substantial, and sustained changes in behavior and perception' (13). As early as 1960, Stace (25) identified several dimensions of mystical experience to include (1) sacredness, (2) noetic quality, (3) deeply felt positive mood, (4) ineffability, (5) paradoxicality, and (6) transcendence of time and space. He further discerned between extrovertive and introvertive mystical experience regarding sense of unity, the difference being that extrovertive involves universal interconnectedness while introvertive entails ego dissolution; the presence of both is considered “complete”. Based on the dimensions identified by Stace, Pahnke and Richards (26) in 1966 developed the Mystical Experience Questionnaire [MEQ]. Mystical experience has often been identified as a phenomenon related to acute psychedelic experience (14), which is defined as a range of subjective effects including visual alteration, acute anxiety, and insightfulness, among others (21), when administered at high dosage (27, 28).

A number of instruments have been designed to measure the intensity of mystical experience, including the [MEQ] (29, 30), Hood Mysticism Scale [HMS] (31), Hallucinogen Rating Scale [HRS] (32), and 5 Dimensions Altered State of Consciousness Questionnaires [5D-ASC] (33), among others. [See Table 1 for details of instruments used.] They measure general psychedelic experience ranging from those attainable at low dose (blissful feeling, positive affect, auditory alterations) to high dose (ego dissolution, transcendence of time and space). Several studies have identified complete or more intense mystical experience as a scoring of MEQ > 0.6 (14, 34). Liechti et al. (35) demonstrated that OBN, one of five subdimensions of 5D-ASC, which includes subdimensions of experience of unity, spiritual experience, blissful state, and insightfulness, has a Pearson correlation of 0.93 with MEQ. According to Roseman et al. (14), then, scoring of OBN > 0.6 can also be used to indicate complete mystical experience.

**Table 1:** Instruments used.

**Questionnaire**	**Acronym**	**Number of questions**	**Factors/Subdimensions**
Mystical Experiences Questionnaire	MEQ	30	Mystical, Positive Mood, Transcendence of Time and Space, Ineffability
Hood Mysticism Scale	HMS	32	Unity, Ineffability, Positive Affect, Sense of Sacredness, Loss of Ego
Hallucinogen Rating Scale	HRS	99	Intensity, Somaesthesia, Affect, Perception, Cognition, Volition
States of Consciousness Questionnaire	SOCQ	100 (43 Pahnke–Richards Mystical Experience Questionnaire; 57 distractors)	Unity, Transcendence of Time and Space, Ineffability, Sacredness, Noetic Quality, Positive Mood
5 Dimensions of Altered State of Consciousness Questionnaire	5D-ASC	96	Oceanic Boundlessness, Dread of Ego Dissolution, Visionary Restructuralization, Auditory Alterations, Altered Vigilance
11 Dimensions of Altered State of Consciousness Questionnaire	11D-ASC	94 (44 scored; 50 distractors)	Experience of Unity, Spiritual Experience, Blissful State Insightfulness, Disembodiment, Impaired Control and Cognition, Anxiety, Complex Imagery, Elementary Imagery, Audio-Visual Synesthesia, Changed Meaning of Perceptions

Sanders and Zijlmans (36) dismiss the concept of mysticism in psychedelic research as non-empirical, while they do support the validity of the three primary instruments (MEQ, HMS, and ASC) used. This was challenged by Breeksema and van Elk (37) on the basis that mysticism as an element of the full human experience, is clinically valid and has been rigorously studied, with multiple well-established instruments. Jylkka (38) agreed with Sanders and Zijlmans that these instruments place specific interpretation on the subjects' experience and are not sufficiently secular, and therefore more neutral instruments are needed. Nonetheless, Jylkka also supported Breeksema and van Elk and added that mystical experience is a valid expression of the patient's worldview. Both Breeksema and van Elk and Jylkka criticize Sanders and Zijlmans for solely valuing biological experience as reductionist.

Olson (39) has suggested that subjective experience may not be necessary for the therapeutic outcome, arguing that an increase in neural plasticity has its own value and would occur independent of the phenomenon; thus, non-hallucinogenic properties of psychedelics may be isolated for use. Nonetheless, Olson goes on to say that the synergy between subjective experience and neurobiological mechanisms may maximize outcome, which was also supported by Yaden and Griffiths (28).

We hypothesize that presence and intensity of the mystical psychedelic experience contributes to therapeutic efficacy, to include both symptom reduction and improved quality of life. This review identifies studies of psychedelic therapy that include data on mystical psychedelic experience and therapeutic efficacy, and explores the possible relationship between the intensity of mystical psychedelic experience and changes in symptomatology as well as quality of life.

While the potential for mystical experience as a mediator of therapeutic efficacy arose incidentally in a systematic review by Romeo et al. (40), the objective of said review was to investigate the biological and clinical markers of the psychedelic response. Andersen et al. (41) reviewed for therapeutic efficacy overall, among several other secondary objectives, for which the role of subjective effects (including mystical experience) was included. This review, in contrast, has as its primary objective the relationship of mystical experience to therapeutic efficacy.

## Methods

### Searches and Study Selection

The study protocol was developed and agreed prior to the review and is registered at PROSPERO, number CRD42021261752. It follows the PRISMA guidelines (42) and checklist [see Supplementary Data Sheet 1]. The literature search was carried out in Embase, MEDLINE, and PsychINFO to include publication dates from January 1990 to August 2021. Search string used was (“psychedelic^*^” OR “psilocybin” OR “LSD” OR “Lysergic acid diethylamide” OR “ayahuasca” OR “DMT” OR “hallucinogen^*^” OR “mescaline” OR “peyote” OR “Dimethyltryptamine” OR “MDMA” OR “MDA” OR “3, 4-Methylenedioxymethamphetamine” OR “Methylenedioxyamphetamine”) AND (“acute-state^*^” or “subjective experience^*^” or “mystical experience^*^” or “mystical-type experience^*^” or “mystical^*^”).

The Multidisciplinary Association for Psychedelic Studies [MAPS] online bibliography was also searched, but as MAPS does not allow Boolean search, the search was conducted differently from above. The word “mystical” was inserted in the fields of both title and abstract; in the keywords field, the following were included (from a pull-down menu which included two misspellings): “subjective effects,” “subjective experience,” “subjecti [sic] effects,” “acute effect,” “acute efects [sic]”, “mystical experience,” “Mystical experiences,” and “Mystical experience”.

Out of 744 articles gained by the above searches, 12 were identified as relevant to this review. The studies included were selected according to the following criteria: (1) clinical trial in design; (2) adult subjects with psychiatric and/or addictive disorders who received psychedelic dosing either in laboratory or clinical setting; (3) subjectively reported data regarding mystical experience and treatment response; and, (4) published in English in a peer-reviewed journal. The reference lists of selected studies and systematic reviews that emerged from the searches were also reviewed for additional studies. Study selection was performed by two independent researchers (hereafter identified as KK and GK).

### Quality Assessment

Newcastle Ottawa Quality Modified Scale (43) was used to assess the quality of open-label uncontrolled studies. Randomized trials were assessed using a Revised Cochrane Risk of Bias Tool for Randomized Trials (44).

Risk of bias [see Supplementary Tables S1, S2] was assessed independently by each of two reviewers according to the following characteristics: blinding, selective outcome reporting, randomization sequence generation, completeness of outcome data, and other sources of bias. First and second reviewers attempted to reach consensus; when not possible, an independent reviewer was consulted. This assessment was performed by primary reviewer (KK) and verified by second reviewer (GK).

### Data Extraction

Data were extracted from each study individually, with a focus on correlation, mediation, and/or prediction between intensity of mystical psychedelic experience and symptomatology decrease, as well as results significance. Also included were participant data (number of participants, age, gender ratio, diagnosis), substance and dose, length of follow-up, instruments, types of statistical analysis, and models of support. This extraction was performed by primary reviewer (KK) and verified by second reviewer (GK).

## Results

### Study Categorization

**Figure 1:**
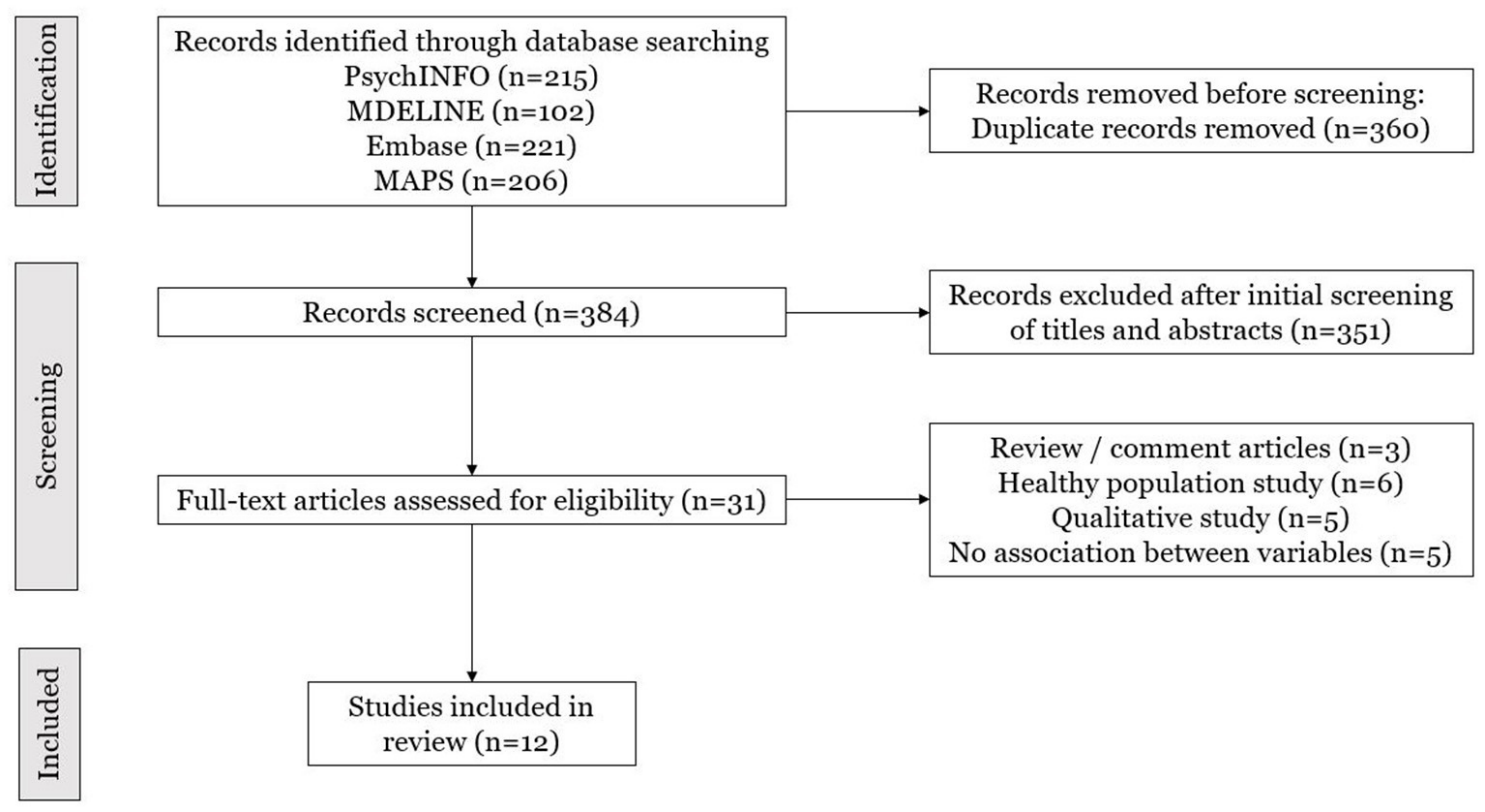
Consort flow chart.

A consort flow chart was developed to depict details of the study selection process, according to the PRISMA guidelines (42) [see Figure 1]. A total of twelve studies were selected based on the inclusion criteria, 6 of which were randomized (12, 23, 24, 45–47) and the other 6 open-label (14, 16, 34, 48–50). Of these studies, five investigated substance use disorder, of which two studies explored Alcohol Use Disorder [AUD] (23, 49), two examined tobacco smokers and were based on the same population dataset (51) but with different follow-up periods (34, 50) and, one inspected cocaine dependency (24). Three studies focused on cancer-related adjustment disorders (12, 45, 46). Four considered depression; Aust et al. (48) investigated Major Depressive Disorder [MDD], while the other three (14, 16, 47) investigated treatment-resistant depression [TRD]. The former two accessed the same population dataset from an open-label feasibility study (52) but with different objectives.

In these studies, three types of drugs were used: psilocybin, ketamine, and ayahuasca, with the former being the most common. Eight studies utilized psilocybin, six of which (12, 34, 45, 46, 49, 50) administered doses ranging from 10 to 30 mg dependent on body mass, with 10 considered low-dose and 30 high. The other two (14, 16) administered 10 and 25 mg doses 7 days apart, irrespective of body mass. Ketamine was used in three studies. Dakwar et al. (24) and Rothberg et al. (23) administered 0.71 mg/kg by 1 infusion over 52 min, and Aust et al. (48) by infusion of 0.5 mg/kg within 40 min, 3 times weekly over a period of 2 weeks. One study (47) investigated ayahuasca, with researchers administering 1 ml/kg in a single oral dosage.

Various models of support were identified pre-, during, and post-treatment. Nondirective support during the treatment was provided in 10 of the 12 studies. Pre- and post-treatment therapies varied or were not utilized. Treatment models included Motivation Enhancement Therapy, Cognitive Behavioral Therapy, eclectic or integrative, and therapeutic rapport with allocated psychiatrists [see Table 2].

**Table 2:** Accompanying model of support.

**Study**	**Therapy Content**		
	**Pretreatment**	**Treatment**	**Posttreatment**
Agin-Liebes et al. (45)*	Eclectic	Nondirective support	Eclectic
Aust et al. (48)	None reported	N/A	N/A
Bogenschutz et al. (49)	MET	Nondirective support	MET
Carhart-Harris et al. (16)**	Therapeutic relationship	Nondirective support	Integrative
Dakwar et al. (24)	None reported	N/A	(follow-up with research staff)
Garcia-Romeu et al. (34)***	CBT	Nondirective support	CBT
Griffiths et al. (46)	Therapeutic relationship	Nondirective support	(follow-up telephone interview)
Johnson et al. (50)***	CBT	Nondirective support	CBT
Palhano-Fonte et al. (47)	None	Nondirective support	(debriefing)
Ross et al. (12)*	Eclectic	Nondirective support	Eclectic
Roseman et al. (14)**	Therapeutic relationship	Nondirective support	Integrative
Rothberg et al. (23)	MET, relaxation technique, mindfulness	Nondirective support	(follow-up phone call)

*MET, Motivational Enhancement Therapy; CBT, Cognitive Behavioral Therapy*.

* Studies with same population.

**Studies with same population.

***Studies with same population.

### Cancer-Related Distress Studies

In two randomized controlled trials [RCT] investigating psilocybin's effect on cancer-related psychiatric distress (12, 46), outcome measures were collected 5 and 6 weeks after the session, respectively, multiple significant correlations were observed between intensity of mystical experience as measured by MEQ 30, administered following session 1, and various measures of symptomatology reduction.

Griffiths et al. (46) reported that out of 20 self-rated primary outcome measures, 18 were reported to have significant correlation (*p* < 0.05) with mystical experience. Individual *p*-values, however, were reported for 4 only, deemed highly significant (*p* < 0.0001). Symptom reduction was indicated according to negative correlations in instruments for depression and/or anxiety, which included Hamilton Anxiety Rating Scale [HAM-A] (*r* = −0.59, *p* < 0.0001), GRID-Hamilton Depression Rating Scale [GRID-HAMD] (*r* = −0.41), Hospital Anxiety and Depression Scale (Total) [HADS T] (*r* = −0.41), The Brief Symptom Inventory [BSI] (*r* = −0.38), HADS (Depression) [HADS D] (*r* = −0.36, *p* < 0.0001), Profile of Mood States (Total) [POMS T] (*r* = −0.35), HADS (Anxiety) (*r* = −0.34), State-Trait Anxiety Inventory (Trait/Anxiety) [STAI T/A] (*r* = −0.31), and Beck Depression Inventory [BDI] (*r* = −0.30). Changes in quality of life were indicated by positive correlations regarding meaningfulness (*r* = 0.77, *p* < 0.0001), spiritual significance (*r* = 0.75, *p* < 0.0001), and life satisfaction (*r* = 0.53, *p*-value not reported, but was identified “significant”) (Persisting Effects Questionnaire [PEQ]); meaningful existence (*r* = 0.41) and quality of life (*r* = 0.32) (McGill Quality of Life Questionnaire [MQOL]); coherence (*r* = 0.41) and death acceptance (*r* = 0.38) (The Life Attitude Profile [LAP-R]); death transcendence (*r* = 0.31) (Death Transcendence Scale [DTS]); and, Purpose in Life (*r* = 0.29). A limitation of this study is the crossover design which didn't allow for double-blind assessment. Additionally, PEQ had not been independently validated at the time of this study.

Ross et al. (12) also identified significant correlation between mystical experience and 4 out of 6 primary outcome measures to include HADS T (Spearman *r* = 0.39; *p* = 0.04), BDI (*r* = 0.49; *p* = 0.01), STAI S (*r* = 0.42; *p* = 0.03), and STAI T (*r* = 0.39; *p* = 0.04). The correlation was further supported by mediation analysis in both studies (statistical data not reported).

Agin-Liebes et al. (45) is a long-term follow-up of Ross et al., conducted at an average of 3.2 to 4.5 years post-psilocybin therapy. Data for mystical experience (measured by MEQ-30), collected on dosing day, did not show any statistically significant correlation with anxiety or depression as measured by HADS, BDI, and STAI. The longer follow-up period of this study, as most are not more than 1 year following treatment, is a particular strength. Limitations of this study include the cross-over design in the parent study, as well as 50% reduction of the original sample size by the time of long-term followup. The researcher's assessment of these limitations is included in the Discussion section.

See Table 3 for details of cancer-related distress studies.

**Table 3:** Cancer-related distress studies.

**Study**	**Population**	**Design**	**Substance and dose**	**N**	**Main results**	**Long-term follow-up**
Agin-Liebes et al. (45)*	Subjects with cancer-related adjustment disorder, anxiety and/or depression features	RCT	Psilocybin (0.3 mg/kg), single dose	15	[Long-term results only]	3.2–4.5 years: No significant correlation between MEQ-30 (post-dosing) and any of the primary outcome measures of anxiety or depression at post-dosing compared to baseline
Ross et al. (12)*	[As above]	RCT	[As above]	29	MEQ-30 (post-dosing) correlated significantly with change in scores of positive therapeutic outcomes at 6 weeks. MEQ total was also a significant mediator of psilocybin administration on therapeutic outcomes.	6.5 months: Changes sustained
Griffiths et al. (46)	Subjects with life-threatening cancer diagnoses and anxiety and/or mood features	RCT	Psilocybin (22 mg or 30 mg/70 kg), single dose	51	Significant negative correlation of MEQ30 (post-dosing) at week 5 with changes in scores of symptomatology and positive correlations with changes in scores regarding quality of life. MEQ30 score as significant mediator of psilocybin administration on therapeutic outcomes.	6 months: Changes sustained

*RCT, Randomized controlled trial; MEQ-30, Mystical Experience Questionnaire*.

*Studies with same population.

### Depressive Disorder Studies

The research in psychedelics for depressive disorders falls under two categories as outlined below.

### Treatment-Resistant Depression

Roseman et al. (14) and Carhart-Harris et al. (16) drew from the same population data of an open-label feasibility study (51), as previously indicated. Both studies considered the dimensions of altered states of consciousness [ASC] as predictor of clinical outcome following psilocybin administration; Roseman et al. (14) administered 5D-ASC while Carhart-Harris et al. (16) utilized 11D-ASC. The absence of a control group due to the open-label design in the original trial is a limitation of both studies.

Roseman et al. found that psilocybin-induced oceanic boundlessness [OBN] positively (standardized beta coefficient = 0.605), and dread of ego dissolution [DED] negatively (standardized beta coefficient = −0.649), predict decrease in depressive symptoms as measured by QIDS-SR (*r*^2^ = 0.59, adjusted *r*^2^ = 0.54). This result was sustained up to 5 weeks post dosing session. Additionally, those who experienced more intense or “complete” OBN (defined by a threshold of OBN > 0.6) demonstrated various improved clinical outcomes of trait anxiety, anhedonia, optimism, and pessimism at intervals of 1-day, 1-week, 5-weeks, 3-months, and 6-months. In the presence of psychedelic-induced acute anxiety, therapeutic outcome was found to be decreased (for details, see Supplementary Table S1 in original source).

Carhart-Harris et al. (16) further focused on OBN, specifically its features of “experience of unity”, “spiritual experience”, “blissful state”, and “insightfulness.” Upon multiple correlation analyses confirming the inter-relatedness between the former three, they were treated as one factor identified as USB (unity, spiritual, blissful). Both mean USB and insight score during 25-mg acute psilocybin experience significantly related to changes in QIDS-SR16 scores at the fifth week (*r* = – 0.49, *p* = 0.03 and *r* = – 0.57, *p* = 0.01, respectively).

In a clinical trial utilizing ayahuasca for TRD (47), no significant correlation was found between Hallucinogen Rating Scale [HRS] and changes in Montgomery-Asberg Depression Rating Scale [MADRS] from baseline to the seventh day, when compared to the placebo group. The ayahuasca group showed significant correlation between changes in MADRS score from baseline to day 7 and HRS subscale of perception (*r* = 0.90, *p* = 0.002). The MEQ-30 sub-dimension of transcendence of time and space correlated with therapeutic outcome (*r* = −0.84, *p* = 0.009), while the other three factors of ineffability, mystical, and positive mood showed no correlations with changes in MADRS score. Additional measures were taken to ensure blindness, which is a strength of this study.

### Major Depressive Disorder

In an uncontrolled open-label ketamine study of adult inpatients with MDD (48), 6 ketamine infusions were given in a 2-week period. Among dimensions of 5D-ASC, applied 4 h after the first infusion, DED was significantly higher among those who did not respond to treatment, specifically the subdimensions of “negative derealization”, “reduced self-control”, and “reduced body control”. In contrast, apart from anxiety, the other 10 dimensions of 11D-ASC showed no significant difference among responders and non-responders. This, therefore, indicates no correlation between treatment outcome and mystical experience. *Post-hoc* study demonstrated a significant negative correlation between openness to new experience and intensity of acute anxiety (*r* = −0.54, *p* < 0.005). The researchers identified the lack of follow-up as a limitation to this study as it didn't allow for outcome of repeated administration. Additional limitations include subjective experience measures administered only after the initial infusion, subjects identified simplistically as responders/non-responders rather than on a spectrum, and a lack of medication control from baseline to final treatment. One strength of this study is its indication of a possible phenotypic response predictor.

See Table 4 for details of depressive disorder studies.

**Table 4:** Depressive disorder studies.

**Study**	**Population**	**Design**	**Substance and dose**	***N***	**Short-term results**	**Long-term follow-up**
Aust et al. (48)	Adult inpatients with MDD	UOLS	Ketamine (0.5 mg/kg over 40 min), three infusions per week for 2 weeks.	31	Significant difference on 5D-ASC subscale ‘anxious ego-disintegration' for responders (≥ 50% MADRS reduction), with no significant difference in the other 4 dimensions after final dose	N/A
Carhart-Harris et al. (16)*	Adult patients with TRD	UOLS	Psilocybin (10 and 25 mg), two doses, 7 days apart	20	Significant association between mean USB score and decrease in QIDS-SR16. USB, dimensions of 11D-ASC, as a significant predictor of clinical outcome at week 5	6 months: Changes sustained
Roseman et al. (14)*	[As above]	UOLS	[As above]	20	From baseline to week 5: significant long-term positive therapeutic outcome (reduction in QIDS-SR16) following higher occurrence and magnitude of OBN and DED OBN, compared to VRS and AUA, is a significantly better predictor of therapeutic outcome “Complete” OBN leads to better clinical outcomes	N/A
Palhano-Fontes et al. (47)	Adult patients with TRD	RCT	Ayahuasca (1 ml/kg), single dose	29	Significant positive correlation between MADRS score changes on day 7 with the HRS subscale 'perception,' and significant negative correlation with the MEQ subscale ‘transcendence of time and space'	N/A

*MDD, Major Depressive Disorder; TRD, Treatment-resistant Depression; UOLS, Uncontrolled Open-Label Study; RCT, Randomized Controlled Trials; 5D-ASC, 5 Dimensions of Altered States of Consciousness Scale; MADRS, Montgomery-Asberg Depression Rating Scale; HRS, Hallucinogenic Rating Scale; USB, Unity, Spiritual Experience, and Blissful state; QIDS, Quick Inventory of Depressive Symptomatology; 11D-ASC, 11 Dimensions of Altered States of Consciousness Scale; OBN, Oceanic Boundlessness; DED, Dread of Ego Dissolution; VRS, Visionary Restructuralization; AUA, Auditory Alterations; VIR, Vigilance Reduction*.

* Studies with same population.

### Substance Use Disorder Studies

Another promising area of psychedelic therapy is substance use disorder, in areas to include alcoholism, nicotine addiction, and cocaine dependence, as well as others.

### Alcohol Use Disorder

Two studies included in this review investigated psychedelics for use in AUD treatment. One study (49) utilized two doses of psilocybin, 4 weeks apart, while the other (23) administered a single dose of ketamine infusion. In both cases, mystical experience was correlated with decrease in drinking.

In the Bogenschutz et al. (49) study, data from both HRS and MEQ was correlated significantly with 4 measures of symptomatology decrease, to include Penn Alcohol Craving Scale (*r* = −0.823, *p* = 0.006, and *r* = −0.810, *p* = 0.008, respectively), Alcohol Abstinence Self-Efficacy Confidence score (*r* = 0.753, *p* = 0.019, and *r* = 0.762, *p* = 0.017, respectively), change in percent of drinking days (*r* = −0.844, *p* = 0.004, and *r* = −0.885, *p* = 0.002, respectively), and percent of heavy drinking days (*r* = −0.763, *p* = 0.017, and *r* = −0.852, *p* = 0.004, respectively), while ASC summary score correlated only with the latter two (*r* = −0.838, *p* = 0.005, and *r* = −0.893, *p* = 0.001, respectively). The researchers acknowledged the absence of biological measures for alcohol use as a limitation. Also discussed was the inability to distinguish the psychedelic effects from that of other therapeutic measures and patient expectancy effects.

Rothberg et al. (23) observed a significant negative correlation between HMS and number of heavy drinking days, defined as (>4 drinks/day for males; >3 drinks/day for females) over the past 7 days, or minimum weekly use of 35 drinks for males and 28 for females (*r* = −0.466, *p* < 0.05). Further, significant correlations were observed between subdimensions of HMS and symptomatology decrease: ineffability and percentage of heavy drinking days (*r* = 0.624, *p* = 0.01); positive affect and percentage of days abstinent (*r* = 0.613, *p* < 0.05); and, positive affect and average number of daily drinks post-infusion (*r* = 0.554, *p* < 0.05). Other than general limitations cited previously, no other limitations were identified for this study.

### Tobacco Addiction

Garcia-Romeu et al. (34) and Johnson et al. (50) drew data from the same population of an open-label pilot study (51); Johnson et al. (50) focused on long-term treatment effects, while Garcia-Romeu et al. specifically analyzed mystical experience as a therapeutic mediator. Smoking cessation, as measured by urine cotinine level, was maintained by 12 of 15 participants to the 6-month interval (34), and by 10 at the 12-month interval (50). Significant predictors of this outcome included higher scores on States of Consciousness Questionnaire (*p* < 0.049), spiritual significance (*p* < 0.047), impact on wellbeing (*p* < 0.043), and personal meaning given to the psilocybin experience (*p* < 0.047) (34). The outcome was also correlated with the MEQ (*r* = −0.55, *p* = 0.03) and personal meaning (*p* < 0.047; *r* = −0.55, *p* = 0.04) (50).

Garcia-Romeu et al. (34) also measured for complete mystical experience; the raw data included 13 (31%) out of 42 sessions categorized as such, 10 (77%) of which were induced during high dose and the remaining 3 (23%) at moderate, by 9 out of 15 participants (60%) during at least one session. The presence or absence of this phenomenon did not necessarily align with smoking cessation and no correlation analysis was made.

Limitations in the pilot study (51) include the self-selection of participants for motivation to quit and ability to participate long-term without financial compensation. A notable finding in this study is that psilocybin, in the relevant context, can generate complete mystical experience at relatively higher rates than in previous healthy volunteer studies.

### Cocaine Dependence

One randomized controlled trial utilizing ketamine infusion was conducted with 18 cocaine-dependent healthy subjects who expressed disinterest in treatment or abstinence (24). Compared to the control group, effects of ketamine included greater mystical experience (HMS), dissociation (Clinician-Administered Dissociative States Scale [CADSS]), and near-death experience phenomena (Near-Death Experience Scale [NDES]). Of these measures, as well as dosage, HMS was found to be the only significant mediator of decrease in symptom of cocaine dependency (β = 0.431, *p* = 0.0175), in that decrease in cocaine use or craving was observed post ketamine administration. A limitation to this study is the researchers' modification of established questionnaires which may have affected the validity of psychoactive effects measures. Another limitation is that certain factors were not included in pre-application assessment which could have had bearing on outcome, to include family history of alcoholism and serum levels.

See Table 5 for details of substance use disorder studies.

**Table 5:** Substance use disorder studies.

**Study**	**Population**	**Design**	**Substance and dose**	**N**	**Short-term results**	**Long-term follow-up**
Bogenschutz et al. (49)	Adult subjects with active alcohol dependence	UOLS	Psilocybin (0.3 mg/kg and 0.4 mg/kg), two doses, 4 weeks apart	9	Quality of ACUTE psychedelic experience (measured by HRS, MEQ and ASC) was a significant predictor of change in drinking behavior at weeks 5–8	6 months: Changes sustained for majority
Rothberg et al. (23)	Adult subjects with active alcohol dependence	RCT	Ketamine (0.71 mg/kg over 52 min), single dose	40	Significant negative correlation between HMS score and number of heavy drinking days. Positive affect subscale significantly correlated with abstinence and daily drinking level	N/A
Garcia-Romeu et al. (34)*	Healthy adult smokers with multiple prior unsuccessful quit attempts, and with a desire to quit smoking	UOLS	Psilocybin (20 mg/70 kg and 30 mg/70 kg), two doses, 2 weeks apart	15	N/A	6 months: Higher scoring on SOCQ, personal meaning, spiritual significance, and impact on wellbeing was a significant predictor of abstinence [quality of life]
Johnson et al. (50)*	[As above]	UOLS	[As above]	15	N/A	12 months: Significant correlation observed between quality of psychedelic session (MEQ; personal meaning) and abstinence
Dakwar et al. (24)	Medically healthy adult subjects with cocaine dependence, disinterested in treatment or abstinence	RCT	Ketamine (0.71 mg/kg over 50 min), sub-anesthetic infusions	18	HMS score was a significant mediator of anti-addictive qualities	

*UOLS, Uncontrolled Open-Label Study; RCT, Randomized Controlled Trials; HRS, Hallucinogen Rating Scale; MEQ, Mystical Experience Questionnaire; ASC, Altered States of Consciousness; PACS, Penn Alcohol Craving Scale total score; AASE, Alcohol Abstinence Self-Efficacy; PDD, percent drinking days; PHDD, percent heavy drinking days; HMS, Hood Mysticism Scale; SOCQ, States of Consciousness Questionnaire*.

*Studies with same population.

## Discussion

This paper has conducted a systematic review of 12 studies in psychedelic administration to an adult population with psychiatric and/or addictive disorders. The association between mystical experience and therapeutic outcome in psychedelic therapy to include ketamine was indicated by most clinical studies in this review. Mystical experience was a significant predictor of improved outcome in several studies (14, 16, 49). Specifically, Roseman et al. (14) found that the complete mystical experience had a direct and strong correlation to improved outcomes. Significant correlation between mystical experience and clinical improvement was established in nine out of twelve studies analyzed for short- and medium-term results (12, 14, 23, 24, 34, 46, 47, 49, 50). Two studies (34, 46) were suggestive of mystical experience as an independent factor in psychedelic-induced therapeutic outcome. This was further supported by the significant mediating effect of mystical experience on outcome (12, 23, 24, 46).

Ross et al. (12) supported the correlation at short-term (6 weeks); however, a follow-up long-term study conducted by Agin-Liebes et al. (45) at 3.2–4.5 years found no significant association remaining between mystical experience and long-term changes. This could be due to the nature of long-term study; participants may have engaged in a variety of other therapies and/or social changes to which they attributed their long-term results. Lack of power due to reduction of sample size by 50% at the long-term followup was also given as a possible explanation.

Subdimensions of mystical experiences have been examined, utilizing instruments such as 5D-ASC, 11D-ASC, HRS, and HMS. The factor of ineffability, cited as particularly impactful, was significantly correlated with symptom reduction (23); this is reflective of the extraordinary quality of mystical experience and its perceived importance. While not a sub-dimension of mystical experience, a high degree of personal meaning attributed to psychedelic experience was associated with symptomatology decrease in two related substance use disorders studies, at long-term followup of 6 months (34) and 12 months (50).

If mystical experience predicts the therapeutic outcome, it is then important to factor in the elements that affect the phenomenon. While mystical experience generally yields positive responses, acute anxiety which can be induced by psychedelics, including dread of ego dissolution, was demonstrated to compromise outcome in trials of psilocybin (14) and ketamine (48), although it did not lead to aggravated symptoms in either study. For optimal results, it is therefore important to establish an environment (or ‘set and setting') in which subjects' anxiety could be minimized; for example, pre-treatment therapy could include relaxation and mindfulness techniques as well as therapeutic rapport establishment (23, 34, 49). Further, Aust et al. (48) demonstrated a significant negative correlation between ketamine-induced acute anxiety and the personality domain of openness to experience.

Other influences on the therapeutic outcome of psychedelics have been proposed in some of these studies, including near-death experience [NDE] (24) and neural plasticity (16). Certain psychedelics, notably ketamine, can induce an NDE type of experience, which shares features with mystical experience of sacredness, ineffability and general sense of transcendence. The profundity of this experience is often perceived as life-changing. In the Carhart-Harris et al. study, psilocybin was considered as a potential catalyst for acute neural plasticity leading to changes in cognition. This may lead to increased personal insight and therefore, symptom reduction. However, according to de Vos et al. (53) causality of psilocybin as a catalyst for neural plasticity cannot be established in human trials.

Limitations of this review include the small number of studies on this subject, generally with small sample size; the study of mystical experience as a therapeutic predictor is still in its infancy, with relatively few studies to date. A possible limitation of the hypothesis is the controversy among scientists regarding the metaphysical nature of mystical experience. Another limitation is that the phenomenon is non-observable and self-reported.

Two common limitations of these studies were relatively small sample size found in all studies, and lack of ethnic, racial, gender, educational, and/or socioeconomic diversity in half (12, 16, 24, 34, 45, 46). The six open-label studies were uncontrolled and by definition, unblinded. A limitation common to the field of psychedelic research is difficulty in achieving double-blinding (47), in that the effects of psychedelics are typically obvious to both subjects and researchers. Models of support represent another limitation, in that it would be challenging to distinguish the effects of psychedelics from that of concomitant therapeutic intervention. Other strengths and limitations are identified by study.

In future studies, these limitations could be addressed as follows. As cited in a number of the reviewed studies, the small sample size and lack of diversity among subjects found in many studies of this nature should be addressed as possible. However, several complications exist. Funding for such studies is lacking, many restrictions including legality apply, and the nature of psychedelics requires a rigorous screening process. Until these issues are addressed the sample size and representation limitations cannot be resolved. In terms of the challenges of blinding, this could be addressed as in the Palhano-Fonte et al. (47) in that the dosing and the follow-up are done by a different team of researchers, and the subjects are naïve to the substance being administered.

In followup to this review, several areas of study are recommended. As this study indicates relationship between mystical experience and therapeutic efficacy, as defined by changes in both symptomatology and quality of life, more studies of an experimental design to assess and possibly establish causality are needed. Assuming the establishment of causality, then study of intensity of said experience becomes necessary; whether intensity has direct bearing on the degree of therapeutic outcome would have to be established, and if so, ways to increase intensity could be explored. This would also lead to research regarding increased dosage and/or the development of new, stronger compounds which maximize this phenomenon while minimizing possible concomitant anxiety.

If causality is established, then the population that would benefit from mystical experience should be identified in detail. This could include study into psychological and/or clinical markers that predispose people to mystical experience and less related anxiety. Examples include age, gender, personality traits, medical and psychiatric history, as well as expectation of, previous experience with, and value placed on the mystical experience itself.

This review hypothesized that presence and intensity of the mystical psychedelic experience contributes to therapeutic efficacy, to include both symptom reduction and improved quality of life. This was clearly indicated in the studies reviewed, in forms of correlation, prediction, and/or mediation. Suggestions for further study have been explored.

## Data Availability Statement

The original contributions presented in the study are included in the article/Supplementary Material; further inquiries can be directed to the corresponding author/s.

## Author Contributions

All authors listed have made a substantial, direct, and intellectual contribution to the work and approved it for publication.

## Funding

This research was supported by the NIHR Biomedical Research Centre (BRC) at South London and Maudsley NHS Foundation Trust and King's College London.

## Author Disclaimer

The views expressed are those of the authors and not necessarily those of the NHS, the NIHR or the Department of Health and Social Care.

## Conflict of Interest

KK is a member of Psychedelic Trials Group PTG at King's College London KCL. KCL receives grant funding from COMPASS Pathways PLC and Beckley PsyTech to undertake phase 1 and phase 2 trials with psychedelics, including psilocybin. GK is an honorary member of PTG at KCL. JR leads the PTG at KCL; COMPASS Pathways PLC has paid for JR to attend trial related meetings and conferences to present the results of research using psilocybin. JR has undertaken paid consultancy work for Beckley PsyTech, Delica Therapeutics and Clerkenwell Health. AC has recently received honoraria for presentations and/or serving on advisory boards from the following pharmaceutical companies: Janssen, Lundbeck, Allergan, and Livanova.

## Publisher's Note

All claims expressed in this article are solely those of the authors and do not necessarily represent those of their affiliated organizations, or those of the publisher, the editors and the reviewers. Any product that may be evaluated in this article, or claim that may be made by its manufacturer, is not guaranteed or endorsed by the publisher.
