# Supplementary material for: Psychedelics, Mystical Experience, and Therapeutic Efficacy: A Systematic Review
Source: Front Psychiatry. 2022 Jul 12;13:917199. doi: 10.3389/fpsyt.2022.917199 (PMC9340494; doi:10.3389/fpsyt.2022.917199)
Supplement: Supplementary file 1 [file Table_1.docx]

| **Study** | **Selection** | | **Outcomes** | | | **Total** |
| --- | --- | --- | --- | --- | --- | --- |
|  | **Representativeness** | **Ascertainment of exposure** | **Assessment** | **Follow up** | **Adequacy of follow up** |  |
| Aust (2019) | **0** | **0** | **1** | **0** | **0** | **1** |
| Bogenschutz  (2015) | **1** | **1** | **1** | **1** | **0** | **4** |
| Carhart-Harris  (2018) | **1** | **1** | **1** | **1** | **1** | **5** |
| Garcia-Romeu  (2014) | **1** | **1** | **1** | **1** | **0** | **4** |
| Johnson  (2017) | **1** | **1** | **1** | **1** | **1** | **5** |
| Roseman  (2018) | **1** | **1** | **1** | **1** | **1** | **5** |

Supplementary Table 1

Risk of Bias Assessment of Uncontrolled Open-Label Studies
