# Supplementary material for: Psychedelics, Mystical Experience, and Therapeutic Efficacy: A Systematic Review
Source: Front Psychiatry. 2022 Jul 12;13:917199. doi: 10.3389/fpsyt.2022.917199 (PMC9340494; doi:10.3389/fpsyt.2022.917199)
Supplement: Supplementary file 2 [file Table_2.docx]

| **Study** | **Random Sequence Generation**  **(Selection Bias)** | **Allocation Concealment (Selection Bias)** | **Blinding of Participants and Personnel (Performance Bias)** | **Blinding of Outcome Assessment (Detection Bias)** | **Incomplete Outcome Data (Attrition Bias)** | **Selective Reporting**  **(Reporting Bias)** | **Other Bias** |
| --- | --- | --- | --- | --- | --- | --- | --- |
| Agin-  Liebes (2020) | **+** | **+** | **+** | **+** | **+** | **+** | - |
| Dakwar (2018) | **+** | **+** | **+** | **+** | **+** | **+** | - |
| Griffiths (2016) | **+** | **+** | **?** | **+** | **+** | **+** | - |
| Palhano-  [Fonte](https://www.ncbi.nlm.nih.gov/pmc/articles/PMC6378413/) (2019) | **+** | **+** | **+** | **+** | **+** | **+** | - |
| Ross (2016) | **+** | **+** | **+** | **+** | **+** | **+** | - |
| Rothberg (2021) | **+** | **+** | **+** | **+** | **+** | **+** | - |
| + low risk of bias  - high risk of bias  ? unclear | | | | | | | |

Supplementary Table 2

Risk of Bias Assessment of Randomised Studies
